# Mastoparan-7 adjuvanted COBRA H1 and H3 hemagglutinin influenza vaccines

**DOI:** 10.1038/s41598-024-64351-7

**Published:** 2024-06-14

**Authors:** Pedro L. Sanchez, Herman F. Staats, Soman N. Abraham, Ted M. Ross

**Affiliations:** 1 Center for Vaccines and Immunology, University of Georgia, Athens, GA USA; 2 Department of Infectious Diseases, University of Georgia, Athens, GA USA; 3 Florida Research and Innovation Center, Cleveland Clinic, Port Saint Lucie, FL USA; 4 Department of Infection Biology, Lerner Research Institute, Cleveland Clinic, Cleveland, OH USA; 5 Pathology Department, School of Medicine, Duke University Medical Center, Durham, NC USA; 6 Department of Immunology, School of Medicine, Duke University Medical Center, Durham, NC USA; 7 Duke Human Vaccine Institute, Duke University, Duke University Medical Center, Durham, NC USA; 8 Department of Molecular Genetics and Microbiology, Duke University Medical Center, Durham, NC USA; 9 Program in Emerging Infectious Diseases, Duke-National University of Singapore, Singapore, Singapore

**Keywords:** Influenza, Vaccine, Adjuvant, Mastoparan, COBRA, Vaccines, Influenza virus

## Abstract

Adjuvants enhance, prolong, and modulate immune responses by vaccine antigens to maximize protective immunity and enable more effective immunization in the young and elderly. Most adjuvants are formulated with injectable vaccines. However, an intranasal route of vaccination may induce mucosal and systemic immune responses for enhancing protective immunity in individuals and be easier to administer compared to injectable vaccines. In this study, a next generation of broadly-reactive influenza hemagglutinin (HA) vaccines were developed using the Computationally Optimized Broadly Reactive Antigen (COBRA) methodology. These HA vaccines were formulated with Mastoparan 7 (M7-NH_2_) mast cell degranulating peptide adjuvant and administered intranasally to determine vaccine-induced seroconversion of antibodies against a panel of influenza viruses and protection following infection with H1N1 and H3N2 viruses in mice. Mice vaccinated intranasally with M7-NH_2_-adjuvanted COBRA HA vaccines had high HAIs against a panel of H1N1 and H3N2 influenza viruses and were protected against both morbidity and mortality, with reduced viral lung titers, following challenge with an H1N1 influenza virus. Additionally, M7-NH_2_ adjuvanted COBRA HA vaccines induced Th2 skewed immune responses with robust IgG and isotype antibodies in the serum and mucosal lung lavages. Overall, this intranasally delivered M7-NH_2_ -adjuvanted COBRA HA vaccine provides effective protection against drifted H1N1 and H3N2 viruses.

## Introduction

Influenza viruses belong to the family *Orthomyxoviridae* and are the cause of annual respiratory infections. Seasonal influenza virus infections can cause severe respiratory disease resulting in hospitalization and death^1^. Antigenic drift and shift allow influenza viruses to escape the host immune defenses by changing the viral surface glycoproteins hemagglutinin (HA) and neuraminidase (NA)^2^. This is important since developing an effective vaccine against ever-changing influenza A viruses (IAVs) becomes difficult since the selected wild-type (WT) strains included in each seasonal influenza vaccine are not always well matched to circulating strains in a given season, thus compromising the effectiveness of the vaccine^3^. Current commercial vaccines often use live-attenuated virus or split-inactivated virus vaccine platforms that induce high titer immune responses^4^. A few of the currently licensed vaccines have been formulated with adjuvants, such as the oil-in-water emulsion MF59^5^. The addition of adjuvants may enhance vaccine induced immune responses in higher risk groups, such as infants with undeveloped immune systems or the elderly with impaired immune responses^6,7^.

Adjuvants serve as pharmacological or biological molecules that enhance the elicitation of specific immune responses upon co-administration with vaccine compounds^8^. To date, most influenza vaccines are administered by parenteral injections^9^. However, an intranasal route of vaccination may induce mucosal and systemic immune responses in order to enhance protective immunity in individuals with weak immune systems^10^. Intranasal vaccines are painless and easier to administer compared to the invasive methods used with injectable vaccines, thus likely improving vaccine uptake worldwide^10^. In this study, Mastoparan 7 (M7-NH_2_) peptides were used as adjuvant with hemagglutinin proteins and compared to hemagglutinin proteins with no adjuvant. The M7-NH_2_ is a 14 amino acid long cationic peptide sequence (INLKALAALAKKIL-NH) derived from wasp (*Vespula lewisii*) venom and has been previously used in mucosal vaccines for enhancing immunity against systemic anaphylaxis caused by peanut allergic responses as well as vaccines that were effective against cocaine challenge, in mice^11–13^. Additionally, M7-NH_2_ enhances the efficacy of a west Nile virus subunit vaccine, administered intranasally in mice^14^. M7-NH_2_ activates mast cells via its Mas-related G-protein coupled receptor member X2 (MRGPRX2)^12,15^. Upon activation of mast cells by M7-NH_2_, there is a release of preformed mediators, such as histamine, proteases, tumor necrosis factor (TNF-α), IL-4 and IL-5 that are degranulated at mucosal surfaces or tissues and may have different functions depending on their location^16^. Additionally, mast cells are activated by cross-linking IgE receptors or IgG Fc gamma IIIA receptors, vital for activating mast cells, and important innate components for mediating the maturation and trafficking of dendritic cells (DCs) to draining lymph nodes^17^. These dendritic cells will activate naïve T cells via TNF-α and other inflammatory mediators, such as IL-1β, IL-33, and IL-18, and thereby modulate the adaptive immune response^16^. Overall, the use of mast cell activators as adjuvants has been shown to be safe, independent of allergic responses, when tested in animal models^12^.

To meet the mission for next-generation influenza vaccines, a computationally optimized broadly reactive antigen (COBRA) design methodology has been used to develop HA and NA molecules that elicit protective broadly-reactive antibodies against drifted influenza virus strains^18,19^. In previous studies, bivalent H1 and H3 COBRA recombinant HA vaccines, B COBRA HA vaccines, H5 COBRA virus-like particle vaccines, or N1 COBRA NA vaccines elicited antibodies with HAI and NAI activity against panels of historical and contemporary influenza A and B viruses in mice, ferrets, and non-human primates, following intramuscular injection which translated to the protection of animals against viral challenge with H1N1, H3N2, H5N1, B-Victoria, and B-Yamagata influenza lineages^3,19–23^. To determine if COBRA designed vaccines were effective following intranasal delivery, mice were administered the COBRA H1 and H3 HA vaccines formulated with M7-NH_2_. Intranasal delivery of these vaccines, when mixed with M7-NH_2_, elicited broadly-reactive serum and mucosal antibodies and protected mice against lethal influenza virus challenge.

## Materials and methods

### Antigen construction and synthesis

COBRA hemagglutinin (HA) proteins corresponding to H1N1 and H3N2 seasonal influenza viruses (IAVs) were design based on the next-generation COBRA methodology as previously described^18^. Briefly, Y2 (H1) COBRA HA was derived from 6232 full length wild-type influenza A(H1N1) HA protein amino acid sequences, residues 1–566 (starting with Methionine as the first amino acid), from human H1N1 virus infections collected from January 1, 2014 to December, 2016 were downloaded from the EpiFlu online database and organized by their date of collection^19^.

For H3 COBRA HA designated, J4 and TJ5, full length wild-type influenza A(H3N2) HA protein amino acid sequences, residues 1–566 (starting with Methionine as the first amino acid), from 54,041 human H3N2 virus infections collected from May, 1 2008 to April 2016 were downloaded from EpiFlu online database and organized by their date of collection. TJ5 was designed using the sequences between May 2008 to September 2012 and J4 was designed using the sequences between May 2013 to April 2016^3^.

Soluble HA proteins were purified from cells transiently transfected into HEK293T with plasmids expressing a truncated HA gene that was cloned into the pcDNA3.1. The truncated HA genes were generated by replacing the transmembrane domain with a T4 fold-on domain, an Avitag, and a 6 × His-tag for purification^24^. The concentration of the soluble HA proteins was determined by conventional bicinchoninic acid assay (BCA) according to the manufacture’s instruction (Thermo Fisher, Meridian Rd., Rockford, IL, USA).

### Vaccination and infection

**Figure 1:**
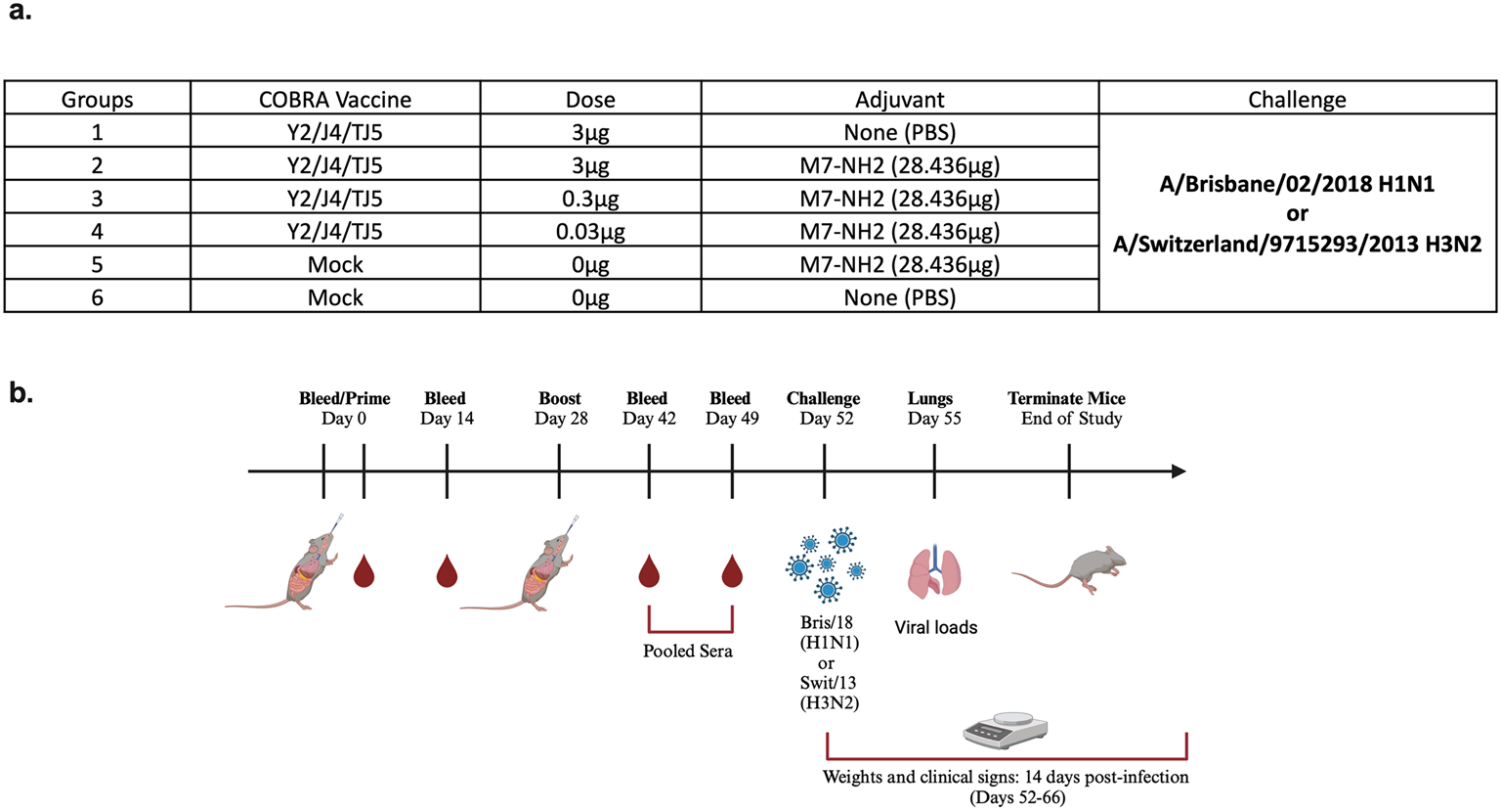
(**a**) DBA/2J (6–8 weeks old) female mice were randomly divided into six groups, with n = 11 mice in each group, and vaccinated intranasally (IN) with 3, 0.3, or 0.03 μg of COBRA HA proteins (Y2, J4, and TJ5) plus the addition of 28.436 μg of the adjuvant, M7-NH_2_ (mast cell degranulating peptide), at a 1:1 ratio. As controls, some mice were vaccinated with COBRA HAs (3 μg) with 0.9% saline, with adjuvant only (0.9% saline plus M7-NH2) or without adjuvant (0.9% saline only). (**b**) Schematic of Study Timeline. DBA/2J mice were bled and prime vaccinated on day 0 follow by blood collection on days 14 and boost-vaccinated on day 28. On days 42 and 49, the mice were bled, followed by intranasal challenge on day 52 with A/Bris/02/2018 or with A/Swit/9715293/2013 viruses. Three days post-infections, lungs were harvested from 3 mice from each group and the remainder mice were monitored for 14 days post infection. Created with BioRender.com.

**Figure 2:**
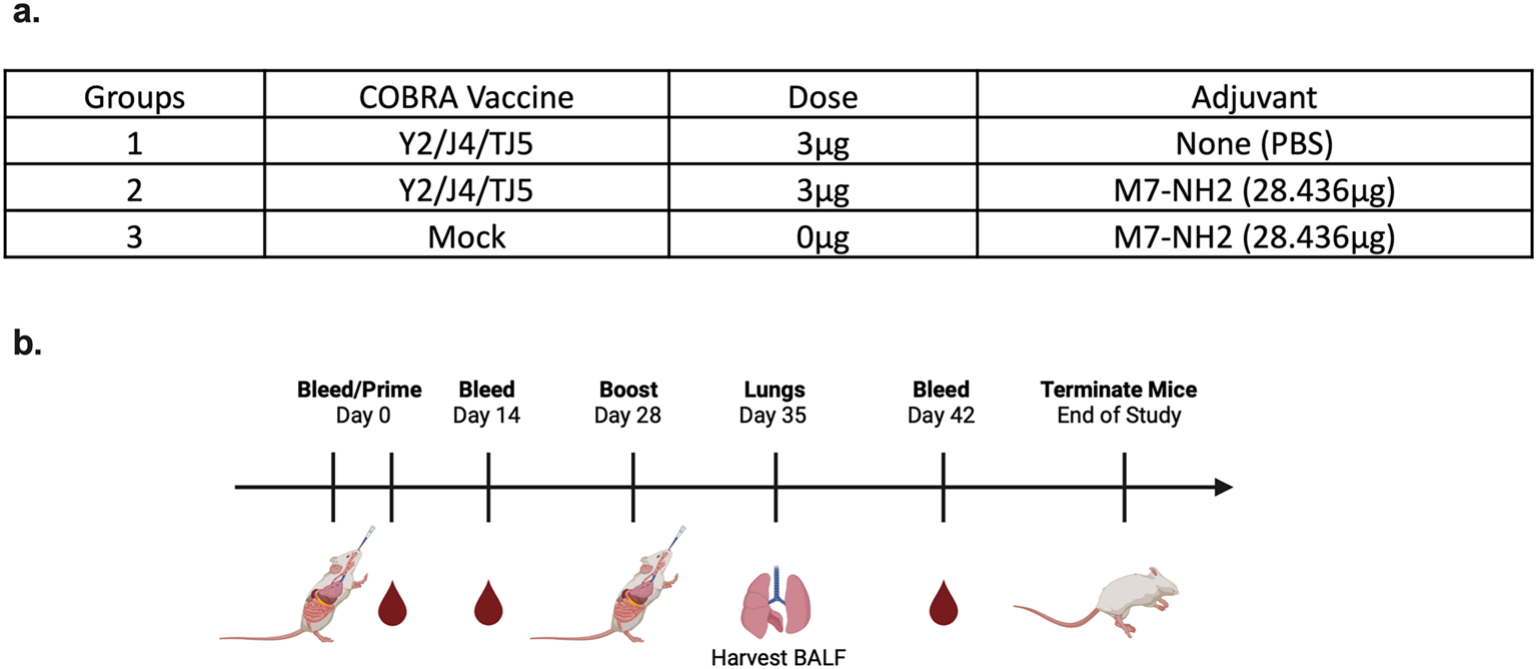
(**a**) BALB/c (6–8 weeks old) female mice were randomly divided into three groups, with n = 10 mice in each group, and vaccinated intranasally (IN) with 3 μg of COBRA HA proteins (Y2, J4, and TJ5) plus the addition of 28.436 μg of the adjuvant, M7-NH_2_ (mast cell degranulating peptide), at a 1:1 ratio. As controls, some mice were vaccinated with COBRA HAs (3 μg) with 0.9% saline, or with adjuvant only (0.9% saline plus M7-NH_2_). (**b**) Schematic of Study Timeline. BALB/c mice were bled and prime vaccinated (IN) on day 0 follow by blood collection on days 14 and 42. On day 28, all of the mice were boost-vaccinated as before. Seven days post-vaccinations (day 35), lungs were harvested from each mouse. Created with BioRender.com.

DBA/2J and BALB/c female mice (n = 66 and n = 30, respectfully; 6–8 weeks old) were purchased from Jackson Laboratory (Bar Harbor, ME, USA) and housed in microisolator cages with access to food and water. The mice were cared for by USDA guidelines for laboratory animals and all procedures were reviewed and approved by the Institutional Animal Care and Use Committee (IACUC) (no. A2020 03–007-Y1-A0 and LRI2935) and performed in accordance with institutional and ARRIVE guidelines. Mice were randomly divided into six (DBA/2J) or three (BALB/c) groups, with n = 11 or n = 10 mice, respectfully, in each group. Prior to vaccination (day 0), all of the mice were bled via the submandibular to confirm seronegative status against seasonal H1N1 influenza viruses, including: Texas/36/1991, Solomon Islands/03/2006 (SI/06), Brisbane/59/2007 (Bris/07, California/07/2009 (Cal/09), Michigan/45/2015 (Mich/15), Brisbane/02/2018 (Bris/18), and H3N2 viruses: Texas/50/2012 (TX/12), Switzerland/9715293/2013 (Swit/13), Hong Kong/4801/2014 (HK/14), Singapore/IFNIMH/2016 (Sing/16), Kansas/14/2017 (KS/17), Switzerland/8060/2017 (Swit/17), and South Australia/34/2019 (SA/19). Mice were anesthetized with 2–3% isoflurane and prime vaccinated intranasally (IN) with 50 μL of vaccine formulations containing 1, 0.1, or 0.01 μg of each of the COBRA rHA proteins, Y2, J4, and TJ5, in cold 0.9% saline solution plus the addition of 28.4 μg of lyophilized M7-NH_2_ powder in cold 0.9% saline solution, with mixing by vortex. As controls, some mice were vaccinated with 50uL of COBRA HA vaccines without adjuvant (0.9% saline only) or mock vaccinated with 50 μL of M7-NH_2_ adjuvant only (0.9% saline plus M7-NH_2_) vaccines (Fig. 1a,b). Upon recovery, all of the mice were place back into their cages and monitored. On day fourteen, all of the mice were bled and sera were separated from blood cells via centrifugation and then stored at − 20  ± 5 °C (Fig. 1b). On day 28, the mice were boosted as before following the same vaccination regimen. Following the boost, all of the DBA/2J mice were bled on days 42 and 49, as before, and the collected sera was separated by centrifugation in microcentrifuge tubes at 10,000 rpm for 10 min, and pooled together equally and then stored at − 20  ± 5 °C. All of the BALB/c mice were boost-vaccinated on day 28, as before, and bled on day 42 and the collected sera was processed and stored as before (Fig. 2a,b). Concurrently, bronchoalveolar lavages were harvested from five BALB/c mice on day 35 post-boost-vaccination. Vaccinated mice were euthanized via carbon dioxide asphyxiation followed by secondarily cervical dislocation and lungs flushed via the trachea with 450uL of cold 1× PBS using 23G needles with 22X1 G polyethylene catheters, attached to 1mL syringes. The samples were placed in sterile microcentrifuge tubes, on ice, followed by centrifugation at 3000×*g* for 5 min. After centrifugation, the supernatants were transferred into fresh sterile centrifuge tubes and stored at − 20 ± 5 °C. At day 52, some of the DBA/2J mice were anesthetized and infected with 8 × 10^6^ PFU of A/Brisbane/02/2018 (H1N1) or with 7 × 10^5^ PFU of the mouse-adapted A/Switzerland/9715293/2013 (H3N2) influenza A viruses (IAV) via intranasal distillation. Upon recovery, the mice were returned to their cages and monitored daily for 14 days post infection for both morbidity and mortality. At day 55, following euthanasia as before, lungs were harvested from 3 DBA/2J mice in each group, snap-frozen on dry ice, and stored at – 80 °C for determining viral lung titers.

### Hemagglutination inhibition assay (HAI)

The HAI assay was performed for the detection of serum antibodies that inhibit binding of influenza viruses from agglutinating red blood cells (RBCs) by preventing binding of viral surface HA to sialic acid residues on RBCs. This protocol was based on the WHO manual for laboratory diagnosis and virological surveillance of influenza^28^. This HAI assay was performed against a panel of H1N1 viruses, including: A/Solomon Island/3/2006, A/Brisbane/59/2007, A/California/07/2009, A/Michigan/45/2015, A/Brisbane/02/2018, and H3N2 viruses: A/Texas/50/2012, A/Switzerland//2013, A/Hong Kong/4801/2014, A/Singapore-IFNIMH-16-0019/2016, A/Kansas/14/2017, A/Switzerland/8060/2017, and A/South Australia/34/2019. The HAI assays were performed as previously decribed^23^. Sera from each mouse was initially treated with receptor destroying enzyme (RDE) (Denka Seiken, Co., Tokyo Japan) for eliminating non-specific inhibitors. In a deep-well, 96-well block, sera was diluted to 1/10^th^ final solutions by reconsituting 100 μL of serum with 3 volumes of RDE in 1 × PBS, followed by overnight incubation at 37 °C. The following day, the RDE-treated sera was heat inactivated in a water bath at 56 °C for 45 min, followed by cooling to room temperature (RT), and the addition of 6 volumes of 1 × PBS. At days 35, lungs from 5 BALB/c mice were harvested and homogenized, in 1mL of cold 1X PBS, using a plunger and 70 μm strainer. In a v-bottom 96-well plate, 25 μL of PBS was added into each well. RDE treated sera or lung homogenate was added in duplicates and serially diluted across the plate. Following serial dilutions, each virus was prepared and tested at a 1:8 solution. The plates were incubated at RT for 20 min for H1N1 influenza viruses or 30 min for H3N2 influenza viruses. Following incubation, 0.8% turkey red blood cells (TRBCs) for H1N1 viruses or guinea pig red blood cell (GPRBCs) for H3N2 influenza viruses were added to all of the corresponding wells, mixed by agiation, and then incubated at RT for 30 min (H1N1) or 1 h (H3N2). After incubation, the titer of each serum and lung sample was reported as the reciprocal dilution of the last well without agglutination. An HAI titer of 1:40 was considered seroprotective as recommeded by the European Medicines Agency Guidelines on Influenza Vaccines^29^.

### Enzyme-Linked immunosorbant assay (ELISA)

To assess total IgG serum antibody reactivity and specificity to COBRA HAs vaccine components or WT IAV HAs, Immulon 4HBX 96-well flat bottom plates (Thermo Fisher Scientific, Waltham, MA, USA) were coated with 100 μL of Y2, J4, TJ5 COBRA or WT Bris/18, Tas/20, or Sing/16 rHAs, at 1 μg/mL in carbonate coating buffer (pH 9.4) and incubated overnight in a humidified chamber at 4°C. Following the incubation, the plates were decanted and blocked with 200 μL, per well, of 4% FBS + 0.05% Tween 20 blocking buffer (BB) in 1 × PBS, for 90 min at 37 °C. During the blocking incubation, serum samples were prepared at 1:100 ratio and serially diluted (1:3) from an initial 1:500 dilution for sera, or prepared at 1:10 ratio for lung lavages, and serially diluted (1:2) from the initial 1:10. Following blocking completion, 100 μL of each sera diluted sample or 50uL of each lung lavage diluted sample was added to the Y2, J4, TJ5 (for sera only) or WT Bris/18, Tas/20, Sing/16 (for sera and lung lavages) rHA coated plates and incubated for 90 min at 37 °C. Plates were washed and 100 μL of prepared secondary goat anti-mouse IgG HRP (Southern Biotech, Birmingham, AL, USA), diluted 1:4000 in BB, added to each well, followed by incubation for 90 min at 37°C. After incubation with secondary antibody, the plates were washed and received 100 μL of 1 × ABTS (VWR Corporation, Matsonford Rd Radnor, PA, USA) solution and incubated for about 13 min at 37 °C. After complete colorimetric development, 50 μL 1% SDS solution was added to each well to stop the colorimetric reaction. The optical density (O.D.) of the samples were immediately read at 414nm in a spectrophotometer (PowerWave XS, BioTek) using the Gen05 software (version 3.14, https://www.agilent.com/en/support/biotek-software-releases) to measure the antibody end-point titers, and compared to positive and negative controls. To further assess IgA and specific IgG1, IgG2a, and IgG2b isotype binding antibodies, samples were processed as before on Y2 coated plates (for sera only) or WT Bris/18, Tas/20, Sing/16 rHAs (for sera and lung lavages) and incubated with secondary goat anti-mouse IgA, IgG1, IgG2a or IgG2b antibody solutions and measured as before. Both sera and lung lavages were prepared at an initial 1:10 and serially diluted (1:2) for detection of IgA and all lung lavages were prepared at an initial 1:10 and serially diluted (1:2) for detection of IgG1, IgG2a, and IgG2b.

### Lung viral titers

MDCK cells were seeded at 1 × 10^6^ cells per 10cm^2^ and incubated for 24h and grown to ~ 95% confluency. Day 55 lungs from each DBA/2J mouse were weighed and homogenized in DMEM supplemented with 1% penicillin–streptomycin (P/S), 10 times their weights. The lung homogenates where then centrifuged at 1500 rpm for 10 min to remove debris and serially diluted, tenfold. Additionally, a tenfold serial dilution of Brisbane/02/2018 or A/Switzerland/9715293/2013 were used as positive controls. The diluted samples were then added to the MDCK monolayers at 100 μL per well, and allowed to infect for one hour, with 15-min shaking intervals, at RT. Moreover, negative control wells received 100 μL of DMEM P/S only. After one hour of infection, the supernatant from each well was aspirated and the wells were washed once with DMEM P/S, with removal of media after the wash. Next, 2mL of a 1:1 solution of 1.6% agarose in 2 × cMEM media containing TPCK-Trypsin at 1 μg/mL was added to each well and allowed to solidify, followed by incubation for 2–5 days at 37 °C with 5% CO_2_. Once cytopathic effects were confirmed, the agarose layers were removed from each well and the cells were fixed with 10% formalin solution for 10 min at RT. After the 10 min, the formalin was removed and the cells were stained with 1% Crystal Violet (Fisher Science Education, Waltham, MA, USA) at RT, for 10–15 min. Following completion of staining, the Crystal Violet was removed and the wells were rinsed in water. The plates were allowed to dry and the plaque forming units (PFUs) were counted, followed by calculation of the lung viral titers as PFU/g of tissue.

### Institutional review board statement

USDA guidelines and regulations for laboratory animals were followed for caring for the mice and all of the procedures performed on the mice were reviewed and approved by the University of Georgia Institutional Animal Care and Use Committee (IACUC) (no. A2020 03-007-Y1-A0 and LRI2935), and performed in accordance with institutional and ARRIVE guidelines.

## Results

### Antigen specific serum and lung binding antibodies

**Figure 3:**
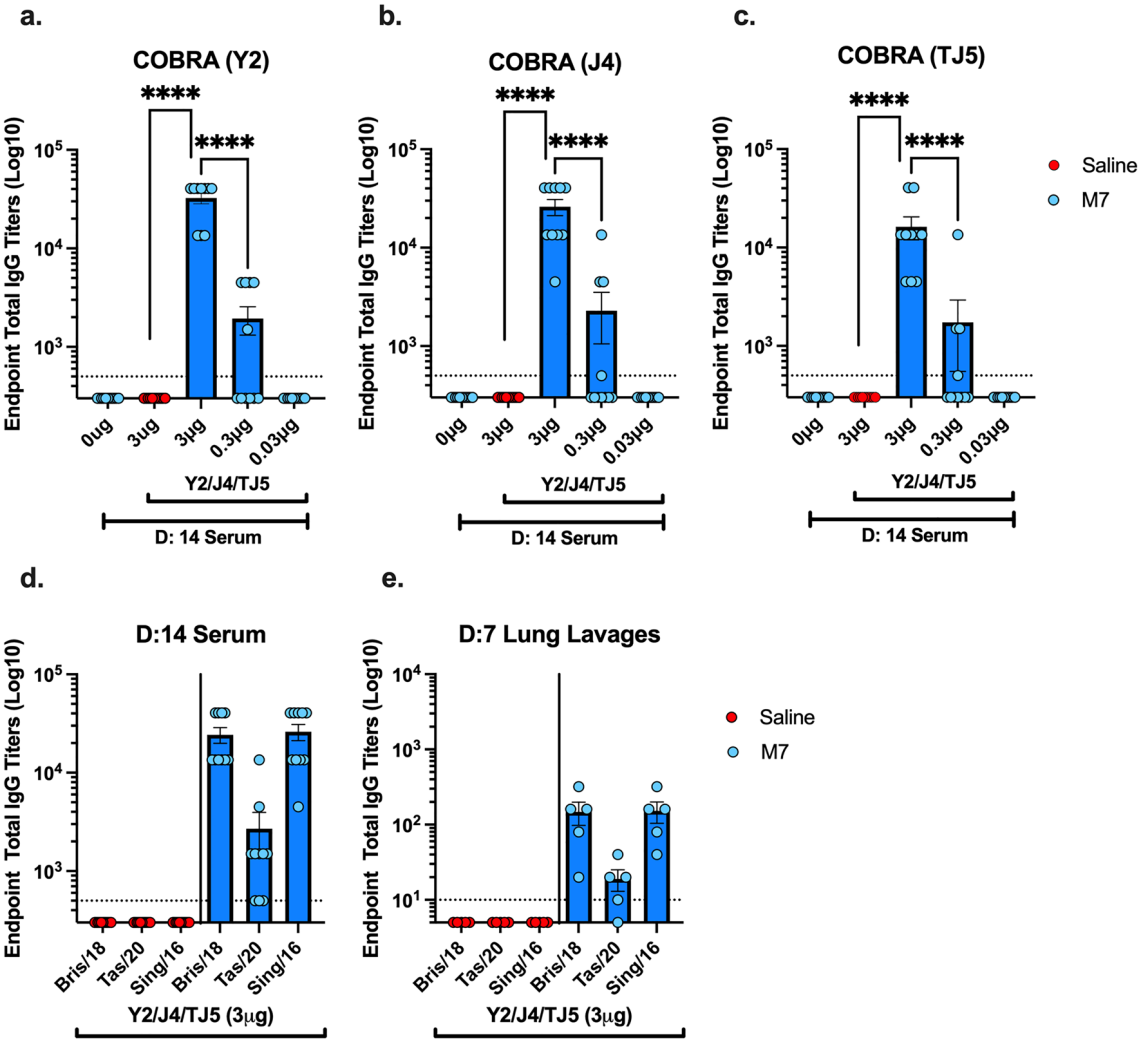
Quantification of total IgG in DBA/2J mice pooled sera collected on days 42 and 49, and in the lung lavages of BALB/c mice harvested on day 35, post two vaccinations. Mice were IN vaccinated with (Red dots) COBRA HAs (3 μg) with 0.9% saline or IN with (Blue dots) M7-NH_2_ (28.436 μg) in the following formulations: with 0.9% saline only, or COBRA HAs (3, 0.3, or 0.03 μg). Antibody responses were measure against plates coated with (**a**) Y2, (**b**) J4, or (**c**) TJ5 COBRA HAs for DBA/2J mice day 42/49 sera, or WT IAV (d and e) Bris/18 H1N1, Tas/20 H3N2, or Sing/16 H3N2 HAs for (**d**) DBA/2J sera and (**e**) BALB/c mice lung lavages. The Y-axis represents the endpoint total IgG titers on a log 10 scale. The X-axis represents the different (**a**–**c**) COBRA HA vaccine formulations (3–0.03 μg) or no COBRA HAs, and (**d**,**e**) WT Bris/18, Tas/20, or Sing/16 HAs. Each column represents dots of 10, 11 or 5 mice per vaccine group and are expressed as the average ± standard error of the mean (SEM). IgG titers were statistically analyzed using nonparametric one-way analysis of variance (ANOVA) by Prism 9 software (GraphPad Software, Inc., San Diego, CA, version 9.4.0, https://www.graphpad.com). A *P* value of less than 0.05 was defined as statistically significant (*P < 0.05; **P < 0.01; ***P < 0.001;****P < 0.0001).

Following the second vaccination, anti-HA IgG binding antibodies to the rHA proteins used for vaccination, WT Bris/18 H1N1, WT Tas/20 H3N2, and WT Sing/16 H3N2 rHAs, was assessed (Fig. 3a–e). Mice vaccinated with adjuvant only or the vaccine only had no detectable IgG antibodies in their sera that bound to any of the rHA proteins. However, all mice vaccinated intranasally with the highest dose of M7-NH_2–_adjuvanted HA (3 μg) had high anti-HA total IgG serum titers against Y2, J4, TJ5, and all of the three WT rHAs proteins (Fig. 3a–e). However, ~ 50% of the mice vaccinated with the COBRA HA (0.3 μg) vaccines plus M7-NH_2_ adjuvant seroconverted and had detectable anti-HA IgG antibodies, whereas no mice seroconverted using the lowest dose of vaccine (0.03 μg) formulated with the M7-NH_2_ adjuvant. Mice vaccinated intranasally with the highest dose of COBRA (3 μg) plus M7-NH_2_ had high anti-HA total IgG titers in their lung lavages against WT Bris/18 and Sing/16 rHAs proteins and 80% of the mice had detectable IgG titers in their lung lavages against WT Tas/20 rHA (Fig. 3e). There were no detectable anti-HA IgA antibodies in the sera of vaccinated mice and few mice had detectable IgA antibodies in the lung lavages.

**Figure 4:**
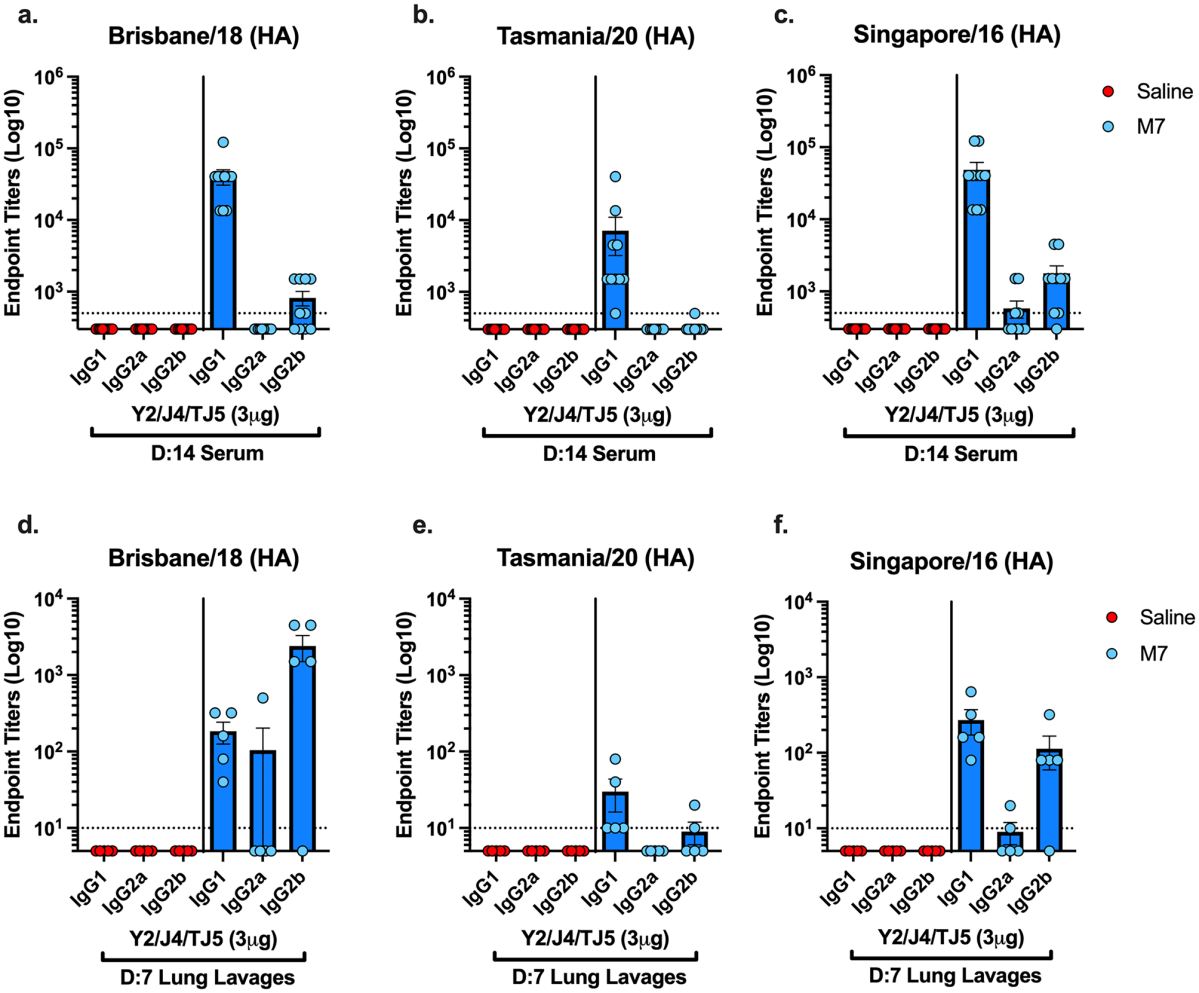
Quantification of IgG1, IgG2a, and IgG2b isotypes in mice sera collected on days 42 and 49, and in lung lavages harvested on day 35, post two vaccinations. Mice were vaccinated IN with (Blue dots) M7-NH_2_-Adjuvanted (28.436 μg) COBRA (3 μg) vaccines or (Red dots) COBRA (3 μg) plus 0.9% saline. Antibody responses were measure against plates coated with (**a**–**f**) WT Bris/18 H1N1, Tas/20 H3N2, or Sing/16 H3N2 HAs. The Y-axis represents the endpoint titers and the X-axis represents the different isotypes. Each column represents dots of 10, 11, or 5 mice per vaccine group and are expressed as the average + /- standard error of the mean (SEM).

Mice vaccinated intranasally with COBRA HA (3 μg) plus M7-NH_2_ had predominantly IgG1 anti-HA serum antibodies with little to no IgG2a or IgG2b antibody isotypes detected (Fig. 4a–c). In contrast, there was significant IgG1 and IgG2b antibodies (1:100–1:1000) in the lung lavages of mice vaccinated with COBRA HA plus M7-NH2 adjuvant (Fig. 4d–f). In comparison, mice vaccinated with COBRA HA proteins alone had no detectable IgG1, IgG2a, or IgG2b in their serum or lung lavages.

### Hemagglutinin-inhibition (HAI) titers from sera and lung Homogenates collected from vaccinated mice

**Figure 5:**
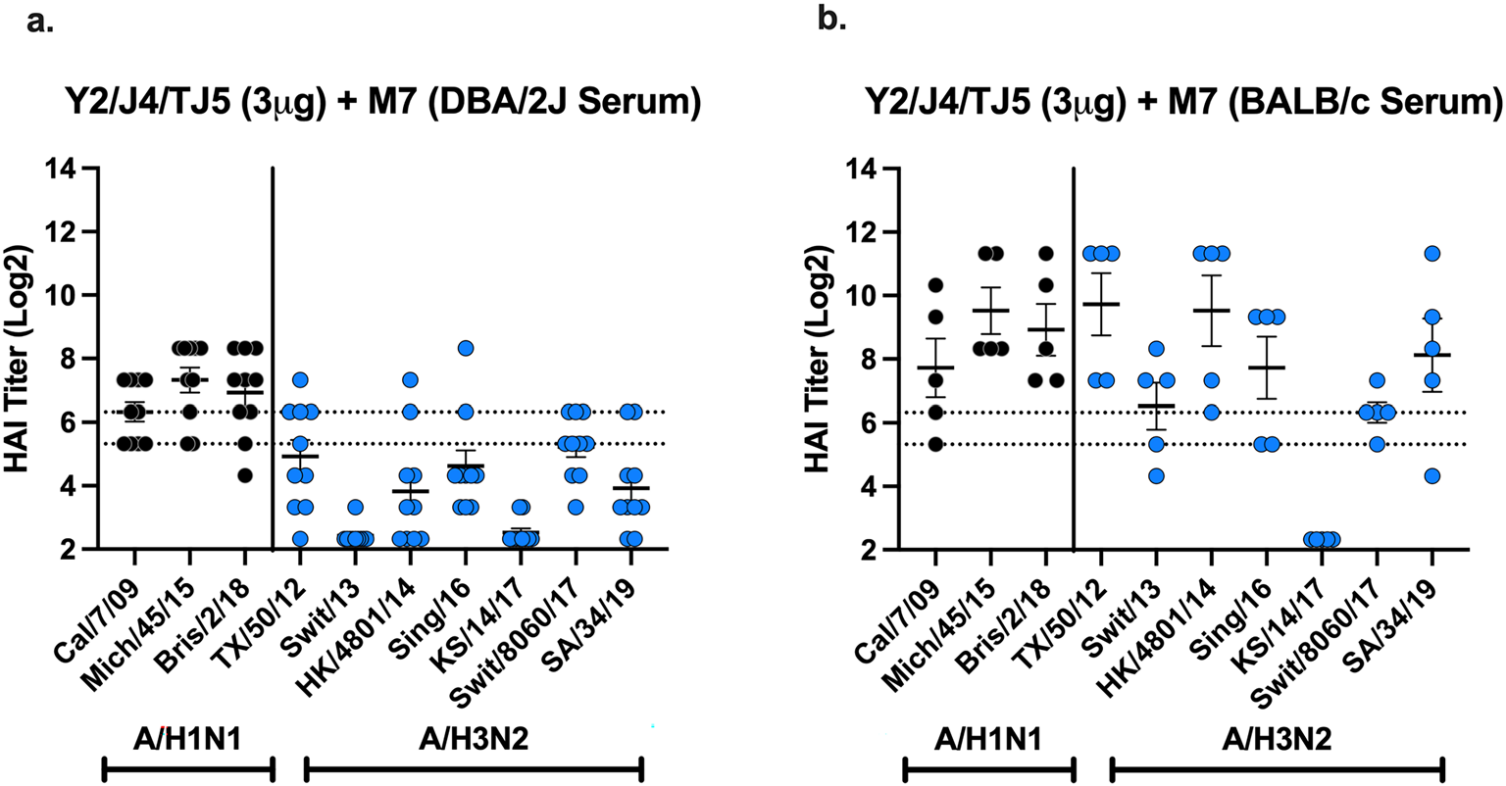
Hemagglutinin-inhibition activity in mice vaccinated with M7-adjuvanted COBRA HA vaccines (Y2, J4, and TJ5). (**a**) Sera collected from individual mice vaccinated with COBRA (3 μg) + M7-NH_2_ (28.436 μg) at days 42 and 49 (DBA/2J) post-boost, was pooled, or (**b**) sera collected at day 42 (BALB/c) post-boost, was tested against a panel of H1N1 (Left) and H3N2 (Right) IAVs. HAI titers (Y-axis) are displayed on a log 2 scale and the panel of H1N1 and H3N2 IAVs are displayed on the X-axis. The two dotted lines represent HAI titers of 1:40 (bottom) and 1:80 (top). Each column represents dots of 10, 11, or 5 mice per vaccine group and are expressed as the average ± standard error of the mean (SEM).

DBA/2J mice vaccinated with 3 μg of COBRA HA (Y2, J4, TJ5) vaccines formulated with M7-NH_2_ had mean HAI titers between 1:80 and 1:160 against the H1N1 influenza viruses. However, all these mice had, on average, HAI titers less than 1:40 (Fig. 5a) against H3N2 viruses. The sera HAI titers of these DBA/2J mice is compared to mice vaccinated with 3 μg of COBRA HA (Y2, J4, TJ5) alone. When these same vaccines were used to vaccinate BALB/c mice, all mice had sera with high HAI activity against the H1N1 viruses as well as 6 of 7 H3N2 influenza viruses (Fig. 5b). The sera HAI titers of these BALB/c mice is compared to mice vaccinated with 3 μg of COBRA HA (Y2, J4, TJ5) alone. There was no detectable HAI activity against KS/17 virus in any of the groups (Fig. 5a,b). There was low or undetectable HAI activity in lung homogenates collected at day 35 from mice vaccinated intranasally with 3 μg of COBRA HA vaccines alone or formulated with M7-NH_2_. In addition, mice vaccinated with lower doses of COBRA HA vaccines (0.3 μg or 0.03 μg) mixed with M7-NH2, M7-NH_2_ adjuvant only, or COBRA HA vaccines (0.3 μg or 0.03 μg) only without adjuvant had sera with no detectable HAI activity.

### *M7-NH*_*2*_*-adjuvanted COBRA HA vaccines protected mice following influenza virus challenge*

**Figure 6:**
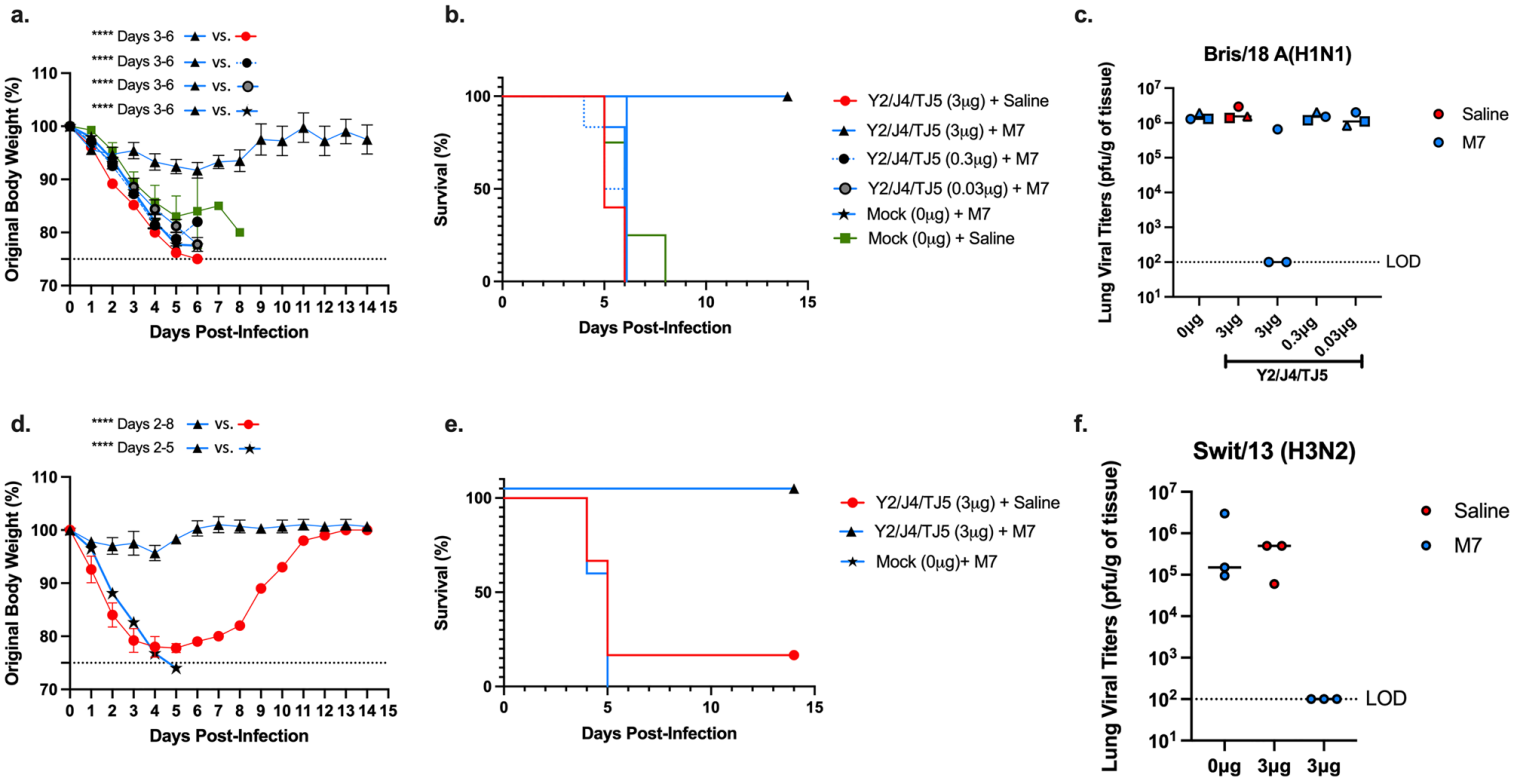
Weight loss, survival, and viral lung titers of DBA/2J mice vaccinated IN with M7-NH_2._ Mice were challenged IN with (**a**–**c**) A/Bris/02/2018 (8 x10^6^ PFU/50 μL) or with (**d**–**f**) A/Swit/9,715,293/2013 (7 x10^5^ PFU/50 μL) and observed for 14 days post-infection. (**a**,**d**) Percent of original body weight, (**b**,**e**) percent survival, and (**c**,**f**) day 55 viral lung titers. The dotted line in panel a represents a 25% weight loss cutoff from the original body weights. The Y-axis in (**a**) represents the original body weights, (**b**) percent survival, and (**c**) the day 3 post-challenge lung viral titers (PFU/g of tissue). The X-axis represents (**a**,**b**,**d**,**e**) the days post-infection and (**c**,**f**) the vaccines with (3–0.03 μg) or without (0 μg) COBRA HAs, adjuvanted with (Blue) M7-NH_2_ (28.436 μg) or (Red) 0.9% saline only. The dotted lines in panels c and f are representative of the limit of detection (LOD). In panel c, the circles represent distinct mice identified as number one in each group, squares represent distinct mice identified as number 2, and triangles represent distinct mice identified as number 3. Viral lung titers were statistically analyzed using nonparametric one-way analysis of variance (ANOVA) by Prism 9 software (GraphPad Software, Inc., San Diego, CA, version 9.4.0, https://www.graphpad.com). A *P* value of less than 0.05 was defined as statistically significant (*P < 0.05; **P < 0.01; ***P < 0.001; ****P < 0.0001).

To assess protection against influenza challenge, vaccinated DBA/2J mice were infected intranasally with the H1N1 virus, Bris/18, or with the H3N2 virus, Swit/13 (Fig. 6). Mice vaccinated with M7-NH_2_ adjuvant only or COBRA HA vaccine alone, and challenged with Bris/18 lost an average ~ 24% of their original body weight and succumbed to infection by day 6 post-infection with no mice surviving infection (Fig. 6a,b). Mice vaccinated with COBRA HA (3 μg) formulated with M7-NH_2_ only lost an average of ~ 8% (day 6) and had 100% survival (Fig. 6a,b). Mice vaccinated with lower doses of the COBRA HA/M7-NH_2_ vaccine lost greater than 18% of their original body weight and all of mice succumbed to infection by day 6 post-infection, (Fig. 6a,b). Mice vaccinated with M7-NH_2_ adjuvant only and challenged with Swit/13 lost an average ~ 26% of their original body weight and succumbed to infection by day 5 post-infection with no mice surviving infection (Fig. 6d,e). Mice vaccinated COBRA HA (3 μg) vaccine alone and challenged with Swit/13 lost an average ~ 22% of their original body weight and succumbed to infection by day 6 post-infection with one mouse surviving infection (Fig. 6d,e). Mice vaccinated with COBRA HA (3 μg) formulated with M7-NH_2_ and challenged with Swit/13 only lost an average of ~ 5% (day 4) and had 100% survival (Fig. 6d,e).

At day 3 following infection with Bris/18 (day 55), mock vaccinated mice, COBRA HA vaccinated only mice, as well as mice vaccinated with the two lowest doses of COBRA HA plus M7-NH_2_ adjuvant, all had high viral lung titers (~ 1 × 10^6^ pfu/g of tissue) (Fig. 6c). In contrast, two of the three mice vaccinated with the highest dose of COBRA HA (3 μg) plus M7-NH_2_ had undetectable viral lung titers (Fig. 6c). One mouse (Fig. 6c; blue square) in the group vaccinated with 3 μg of COBRA rHA plus M7-NH_2_ adjuvant had viral lungs titers (6.55 × 10^5^ pfu/g of tissue). While this mouse had anti-HA IgG endpoint dilution serum titer of ~ 1 × 10^4^ against WT Bris/18 rHA, this mouse had greatly reduced HAI titers (1:20) compared to the other 2 mice in the group (Fig. 6c and Table 1). However, this particular mouse also had little weight loss (98% of original weight at day 3 post-infection; Fig. 6a; Table 1), following viral challenge. The other 2 mice pertaining to this vaccine regimen both had high HAI titers (1:320) and total endpoint IgG titers of 1.4 × 10^4^ against WT Bris/18 rHA (Table 1). There were also no detectable viral lung titers (Fig. 6c and Table 1) or visible plaques at day 3 post-infection (Suppl. Fig. S1b). Moreover, mice vaccinated IN with M7-NH_2_ alone, COBRA HA (3 μg) alone, or lower doses of vaccine mixed with M7-NH_2_ had significant weight loss (p < 0.0001) (Fig. 6a) with no mice surviving challenge (Fig. 6b), and high viral lung titers (~ 1 × 10e + 6 pfu/g of tissue) at day 3 post-infection (Fig. 6c, Table 1, and Suppl. Fig. S1). Following challenge with Swit/13 (day 55), mice vaccinated with COBRA HA (3 μg) vaccines alone had between 6.4 × 10^4^ and 5 × 10^5^ pfu/g of tissue (Fig. 6f). Mice vaccinated with M7-NH_2_ alone had between 9.4 × 10^4^ and 3 × 10^6^ pfu/g of tissue (Fig. 6f). On the contrary, mice vaccinated with COBRA HA (3 μg) plus M7-NH_2_ had undetectable viral lung titers (Fig. 6f).

**Table 1:** Correlation of immunology and pathology pre- and post- Bris/18 IAV infection in DBA/2J mice.

Bris/18 HAI titers		Y2/J4/TJ5				Mock + saline
Mouse ID	Mock + M7	3 μg + Saline	3 μg + M7	0.3 μg + M7	0.03 μg + M7	
1	1:5	1:5	1:320	1:5	1:5	1:5
2	1:5	1:5	1:20	1:5	1:5	1:5
3	1:5	1:5	1:320	1:5	1:5	1:5
End-point total IgG titers in serum against WT Bris/18 rHA						
1	0	0	1.4 × 10^4^	0	0	0
2	0	0	1.4 × 10^4^	0	0	0
3	0	0	1.4 × 10^4^	0	0	0
% weight loss at day 3 post-infection						
1	10	17	0	14	16	13
2	13	17	2	10	15	16
3	14	16	1	7	15	13
Viral lung titers (PFU/g of tissue)						
1	1.29 × 10^6^	2.95 × 10^6^	0	1.50 × 10^6^	2.00 × 10^6^	1.35 × 10^6^
2	1.31 × 10^6^	1.39 × 10^6^	6.55 × 10^5^	1.19 × 10^6^	1.10 × 10^6^	4.25 × 10^6^
3	1.94 × 10^6^	1.54 × 10^6^	0	2.06 × 10^6^	8.35 × 10^5^	1.12 × 10^6^

1 = circles, 2 = squares, 3 = triangles.

Comparison of HAI titers against H1N1 Bris/18 IAV and endpoint of total IgG against Bris/18 IAV in DBA/2J mice sera after boost vaccination, and prior to influenza infections. Also represented are percent weight loss and viral lung titers of the corresponding mice from each vaccination group, post-infection with Bris/18. The data points represent mouse 1 (circles), mouse 2 (squares), and mouse 3 (triangle) from each vaccine group. The shapes are identifiers for Fig. 6c.

## Discussion

Most seasonal influenza vaccines are not formulated with an adjuvant^30^. Although adjuvants are tolerable when administered to individuals, they may be associated with increased inflammation and reactogenicity, particularly at the injection site^6^. However, adjuvanted influenza vaccines have been approved in the U.S. and European Union (E.U.) to increase the effectiveness of vaccines for the elderly^30^. MF59 is a squalene oil-in-water adjuvant added to the split inactivated influenza vaccine (marketed under the name Fluad by Sequiris, Holly Springs, NC, USA)^31^. The next-generation of adjuvants for influenza virus vaccines are currently being tested by many groups which include toll-like-receptor (TLR) agonists such as GLA (glucopyranosyl lipid A) (TLR4) and CpG oligodeoxynucleotides (TLR9), or saponins (Iscomatrix)^32^. There are many challenges when tailoring an adjuvant to a vaccine of interest in order to stimulate the necessary protective immune responses. Stimulating the innate immune system will modulate the adaptive immune response and is an efficient mechanism for eliciting protective immunity against viral infection^33^. Eliciting specific immune responses depends on the type of adjuvant used since some adjuvants induce T-bet and STAT-4 signaling pathways that lead to polarized responses that are skewed towards a pro-inflammatory Th1 response and other adjuvants result in induction of STAT-6 and GATA-3 signaling pathways that drive responses towards an anti-inflammatory Th2 response^34,35^. Additionally, adjuvant physicochemical properties and formulations play important roles for how an adjuvant interacts with vaccine components. To date, most adjuvants are formulated with injectable vaccines and administered intramuscularly^36^. An intranasal route of vaccination may induce mucosal and systemic immune responses in order to enhance protective immunity in individuals with weak immune systems, and may be easier to administer compared to invasive injectable vaccines since they do not require needles^36^.

In this study, M7-NH_2_ was administered intranasally with COBRA HA proteins in a dose-dependent approach. Vaccinated mice had high titers of blocking antibodies in their sera after two vaccinations against panels of H1N1 and H3N2 influenza viruses. Following vaccination, influenza virus-specific IgG antibodies are induced after memory B-cell activation in the spleen that further differentiate into serum IgG isotypes^37^. In the presences of T helper (Th) type 1 cytokines, such as IFN-γ, isotype class switching results in IgG2a secreted antibodies^38^. In contrast, in the presence of Th2 cytokines, such as IL-4 or IL-5, IgG isotype switching results in IgG1 secreted antibodies^14,39^. Although Th1 pro-inflammatory immune responses are ideal for clearance or prevention of influenza virus infection, a simultaneous Th2 anti-inflammatory response may be beneficial for counteracting and depolarizing detrimental local and systemic outcomes caused by increased levels of inflammation. Mice vaccinated with COBRA HA proteins plus M7-NH_2_ had high titer IgG1 serum antibodies against three WT H1N1 and H3N2 IAV HAs, with some level of detectable IgG2b antibodies, but little to no IgG2a. These finding are in agreement with previous studies demonstrating comparable enhancement of IgG1 with minimal IgG2a, in mice vaccinated with M7-NH_2_-adjuvanted subunit vaccines, thus indicating a Th2 polarized response induced by M7-NH_2_^14^.

Notably, mice vaccinated intranasally with COBRA HA antigens plus M7-NH_2_ had total serum anti-HA IgG titers ~ 10^4^–10^5^ (primarily IgG1) compared to mice vaccinated intranasally with M7-NH_2_ alone or the COBRA HA vaccine alone. Vaccinating intranasally using M7-NH_2_ has the potential to induce protective antibodies in both the nasal mucosal cavity (local) and in sera (systemic). In the sera, vaccine elicited anti-HA serum IgG, IgG1, and IgG2a antibody titers were similar to the levels of anti-HA antibody titers in the lung lavages of mice, with little to no IgA antibodies. These finding are in contrast to the intramuscularly administered AddaVax, an MF59-like adjuvant, which does not induce measurable mucosal responses in young or old mice vaccinated mice^40^.

Serum HAI titers ≥ 1:40 in people following vaccination with commercial seasonal influenza vaccines are seroprotective in at least 50% of the vaccinated population and are used as a standard of an effective influenza vaccine in people^29^. Sera and lung lavages collected from mice vaccinated with COBRA HA antigens (3 μg) plus M7-NH_2_ had seroprotective antibody titers against a panel of H1N1 viruses isolated from 2009 to 2018. These same serum samples had a lower average of HAI activity against H3N2 viruses isolated between 2012 and 2019 in DBA/2J mice but still elevated in BALB/c mice. However, there were mice with low HAI titers that still survived viral challenge without significant weight loss. The dominating epitopes on the head region of HA of the wild-type and COBRA H1 subtypes lead to the recognition and binding by antibodies directed toward the HA head. COBRA HA proteins could potentially elicit antibodies directed towards the conserved stem region, and thus could contribute toward protection, independent of HAI activity^41^. Additionally, some HAI activity against three H3N2 IAVs was observed in lung homogenates of mice vaccinated with COBRA vaccines, suggesting that protective antibodies could also be retained within mucosal tissue. However, IN administration of COBRA HA proteins with M7-NH_2_ did elicit HAI titers against both H1N1 and H3N2 influenza viruses, systemically and in the nasal respiratory tract. Mice vaccinated IN with COBRA HA antigens (3 μg), adjuvanted with M7-NH_2_, survived Bris/18 (H1N1) virus challenge, with only about ~ 8% weight loss at day 6 post-infection that may correlate with the slightly lower HAI titers against the challenge strain in mouse number 2. HAI titers could potentially be enhanced following an additional vaccine boost. Additionally, mice vaccinated IN with COBRA HA antigens (3 μg), adjuvanted with M7-NH_2_, survived Swit/13 (H3N2) virus challenge, with only about ~ 4% weight loss at day 4 post-infection.

While other adjuvants such as aluminum salts, MF59, adjuvant system 03 (AS03), or an alternative adjuvant system of AS03 (AF03) may be compatible with intramuscularly administered influenza vaccines, many gaps remain in terms of their mechanism of action. To date no intranasal adjuvants have been approved for use with influenza virus vaccines. This in turn poses limitations and challenges in terms of inducing the necessary immune responses in mucosal compartments that are exposed to the external environment, where respiratory pathogens, such as influenza viruses, invade the host.

In general, rHA COBRA HA vaccines alone administered intranasally in mucosal compartments without M7-NH_2_ adjuvant provide little to no immunogenicity. In this study, even with very low doses of vaccines, using the same amount of M7-NH_2_ adjuvant failed to be immunogenic. To address this challenge, and to tailor this specific adjuvant to the COBRA HA vaccines for comparing to unadjuvanted COBRA HA vaccines, and explore the adjuvant-enhanced effect on vaccine efficacy, this dose–response study allowed for identifying the optimal dose of COBRA HA (3 μg) vaccines required for formulating with M7-NH_2_ and successfully enhance the immunogenicity of the antigens, when administered intranasally.

The findings in this study demonstrated that M7-NH_2_ mast cell degranulating peptide adjuvant, was able to successfully enhance the immunogenicity following intranasal COBRA (H1/H3) HA vaccination in mice and reduce morbidity and mortality following challenge with H1N1 and H3N2 influenza viruses. Herein, M7-NH_2_ peptides have potential as an intranasal adjuvant that induces mucosal and systemic immune responses to enhance protective immunity in individuals with weak immune systems, such as young kids and older adults, and provide for easier administration compared to injectable vaccines. Moreover, adjuvanting COBRA HA vaccines with M7-NH_2_ peptides could potentially enhance the immune responses in humans, thus allowing for assessment of seroconversion in sera collected from participants in future human clinical trials.

### Supplementary Information

Supplementary Figure 1.

## Data Availability

The data are contained within the article and supplemental materials.
