# Supplementary figures and images for: Mastoparan-7 adjuvanted COBRA H1 and H3 hemagglutinin influenza vaccines
Source: Sci Rep. 2024 Jun 14;14:13800. doi: 10.1038/s41598-024-64351-7 (PMC11178843; doi:10.1038/s41598-024-64351-7)

## Slide 1
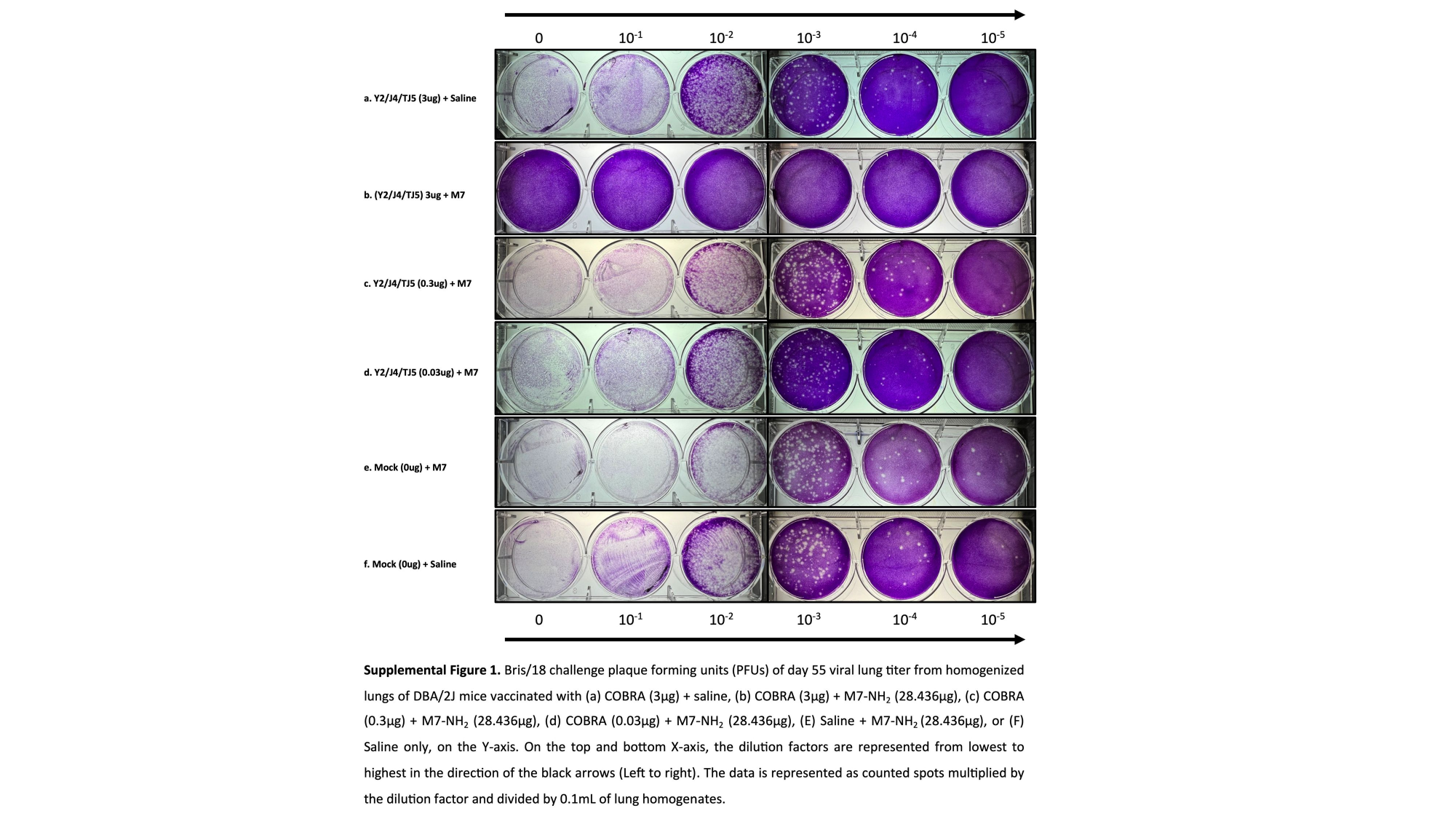

Supplement: Supplementary file 1 — Supplementary Figure 1. [file 41598_2024_64351_MOESM1_ESM.pptx]
